# Management of Locally Advanced Esophageal Cancer

**DOI:** 10.31729/jnma.4299

**Published:** 2021-04-30

**Authors:** Binay Thakur, Mukti Devkota, Manish Chaudhary

**Affiliations:** 1 Department of Surgical Oncology, BP Koirala Memorial Cancer Hospital, Bharatpur, Nepal

**Keywords:** *combined modality therapy*, *esophageal cancer*, *esophagectomy*

## Abstract

Esophageal cancer is diagnosed usually at a locally advanced stage. Surgery alone has less optimal results and a multimodality approach has been established as the standard of care for cII-III stages of esophageal cancer. This review focuses on the recent evidences of management of esophageal cancer with various variations in approaches in Eastern and Western countries. The major difference is the selection of induction treatment. Till the results of some ongoing trials become available, most of the evidences support neoadjuvant chemoradiation followed by surgery for squamous cell carcinoma and perioperative chemotherapy and surgery for adenocarcinoma.

## INTRODUCTION

Esophageal cancer is considered as one of the most aggressive of gastrointestinal malignancies. The overall 5-year survival rate ranges from 15 to 25%.^1^ At diagnosis, nearly 50% of patients have cancer extending beyond the locoregional confines of the primary and 70%-80% of the resected specimens harbor metastases in the regional lymph nodes.^2^

The two major subtypes of esophageal cancer-squamous cell carcinoma (SCC) and adenocarcinoma (AC) have different geographical distribution. SCC has a higher prevalence in East Asia, Eastern and Southern Africa, and Southern Europe, whereas AC is prevalent in North America and other parts of Europe.^3^

Esophagectomy has always remained the mainstay of treatment for esophageal cancer.^4^ But it has been already established that for locally advanced esophageal cancer, surgery alone gives poor results and a multidisciplinary approach needs to be provided.^5^ Here, we review the recent update in the management of clinically locally advanced esophageal cancer (cII-III).

## TREATMENT MODALITIES

The main modality of curative treatment includes:

1. Neoadjuvant chemotherapy followed by surgery
2. Perioperative chemotherapy and surgery
3. Neoadjuvant chemoradiation followed by surgery
4. Definitive chemoradiation.
5. Definitive chemoradiation and salvage surgery

## NEOADJUVANT CHEMOTHERPY

### Squamous cell carcinoma (SCC)

The JCOG9907 trial compared preoperative chemotherapy (cisplatin + 5-FU) followed by esophagectomy versus esophagectomy followed by postoperative chemotherapy for patients with clinical stage II/III SCC (excluding T4 lesions).^13^ Three hundred and thirty patients were randomized to either preoperative or postoperative chemotherapy with 2 cycles of cisplatin and 5-FU. Progression-free survival and overall survival were significantly better in the neoadjuvant group. Based on these results, neoadjuvant chemotherapy with cisplatin + 5-FU is the current standard treatment for cII/III SCC in Japan. For patients who undergo upfront surgery, postoperative chemotherapy is recommended if the pathologic examination detects lymph node metastasis.^12^

In contrary to eastern perspective, West does not consider neoadjuvant chemotherapy as a standard treatment modality for SCC.

### Adenocarcinoma

Most of the treatment guidelines of neoadjuvant treatment protocols for adenocarcinoma cII-III come from west. There are three landmark randomized trials which need attention.

MAGIC trial^14^:

Cisplatin, 5-FU and Epirubicin (ECF) based chemotherapy was administered preoperatively and postoperatively (3 cycles each). Enrolled patients included predominantly patients with gastric cancer; however, there was a subgroup of patients with esophagogastric junction and esophageal cancer. Perioperative chemotherapy improved DFS and OS (P< .0001), with 5-year OS of 36% in perioperative chemotherapy versus 23% in surgery alone.

Federation National des Centers de Luttre contre le Cancer (FNCLCC) trial^15^:

224 patients of surgically resectable distal third esophageal, gastroesophageal junction (GEJ), or gastric AC were enrolled, with 113 randomized to perioperative chemotherapy and 111 to surgery alone. Chemotherapy patients underwent 2 to 3 cycles of neoadjuvant cisplatin + 5-FU, followed by surgery 4 to 6 weeks after, and 3 to 4 cycles of adjuvant therapy. Surgery in both groups consisted of resection of the tumor to adequate margins with extended lymphadenectomy, with the approach being surgeon-dependent. Approximately 75% of patients in each group had distal esophageal/GEJ tumors. OS was improved for patients undergoing perioperative chemotherapy over surgery alone (P = .02). Patients undergoing perioperative chemotherapy alone had a higher rate of DFS (38% vs 19%, P = .01) and R0 resection rate (87% vs 74%, P = .004).

Fluorouracil, Leucovorin, Oxaliplatin, and Docetaxel (FLOT 4) trial^16^

FLOT 4 study randomized 716 patients with gastric or GEJ AC (T2 or N+, staged by cross sectional imaging and endoscopic ultrasound) to perioperative Epirubicin/Cisplatin/Capecitabine (ECX) or ECF given in 3 neoadjuvant and 3 adjuvant settings, Orfluorouracil/ Leucovorin/Oxaliplatin/Docetaxel (FLOT) given in 4 neoadjuvant and 4 adjuvant settings. 360 patients were assigned to ECF/ECX and 356 patients to FLOT. Surgery was scheduled 4 weeks after the last cycle of preoperative chemotherapy. Ivor-Lewis with two field lymphadenectomy (2-FD) was considered for GEJ type I whereas extended total gastrectomy with D2 dissection for GEJ II/III lesions. Overall survival was increased in the FLOT group compared with the ECF/ ECX group (hazard ratio [HR] 0.77; 95% confidence interval [CI; 0.63 to 0.94]; median overall survival, 50 months [38.33 to not reached] vs 35 months [27.35 to 46.26]).

### NEOADJUVANT CHEMORADIATION (nCTRT)

The older randomized trials for SCC form eastern countries did not show any difference in survival except for trial by An, et al. and Cao, et al. from China.^17-20^ Yang and colleagues^21^ recently reported the results of phase III randomized clinical trial (NEOCRTEC5010) comparing nCTRT followed by surgery versus surgery alone. Four hundred fifty one patients with locally advanced SCC (T1-4N1M0/T4N0M0) were enrolled. The nCTRT group received 2 cycles of Vinorelbine and Cisplatin and a total of 40.0 Gy of radiation in 20 fractions. nCTRT resulted in 43.2% pCR, a superior R_0_ resection rate (98.4% vs 91.2%), improved median survival (100.1 months vs 66.5 months), disease-free survival (100.1 months vs 41.7 months), and 3-year overall survival (69.1% vs 58.9%). In a multivariate analysis, preoperative chemoradiation was an independent factor in improved overall survival.

The landmark trial that has established nCTRT as the standard practice for the treatment of resectable esophageal cancer is the Chemoradiotherapy for Oesophageal Cancer Followed by Surgery Study (CROSS) trial.^22^ Patients with both SCC and AC of T1N1M0 or T1-3N_0_-1M_0_ were randomized to nCTRT (n = 178) and to surgery (n = 188) alone over a period of 4 years. Of the 366 patients analyzed, 75% had AC, 23% had SCC, and 2% had undifferentiated cancers. In nCTRT arm, weekly Carboplatin and Paclitaxel for 5 weeks in combination with radiation (41.4 Gy in 23 fractions) was given. R0 resection was significantly higher in the nCTRT group (92% vs 69%; P < .001). Of those who completed chemoradiation, pCR was 29%. pCR rate was significantly higher in patients with SCC compared with esophageal AC (49% vs 23%; P= .008). Nodal involvement was also significantly lower in nCTRT group compared with the surgery-alone group (75% vs 31%; P<. 001). Median overall survival was significantly higher in the multimodality group (49.4 months vs 24 months; HR, 0.66; P = .003). Five-year survival was also higher in the multimodality group (47% vs 34%; HR, 0.66; P = .003). Shapiro and colleagues,^23^ in an extended median follow-up to 84 months again illustrated better results favoring nCTRT arm. Median survival for SCC was 81.6 months for the multimodality group versus 21.1 months for the surgery-alone group (HR, 0.48; P = .008). Median survival for AC was 43.2 months versus months (HR, 0. 73; P = .038).

To date, Japanese surgeons have been reluctant to accept the superior results of nCTRT from Western data. their superior outcomes may be caused by the differences in their patient population tumor biology and their surgical techniques. In order to study the multimodality therapy with the best efficacy for their patient population, Japanese investigators have initiated a 3-armed phase III randomized trial (JCOG1109) comparing the standard preoperative chemotherapy (cisplatin, 5-FU) with an enhanced preoperative chemotherapy (docetaxel, cisplatin, 5-FU) and a preoperative chemoradiation regimen (cisplatin, 5-FU plus radiation).^24^ Hopefully, this trial will resolve the issue of preoperative chemotherapy vs preoperative chemoradiation.

### DEFINITIVE CHEMORADIATION (dCTRT)

In East, Definitive chemoradiation is considered for patients with unresectable disease, patients who are poor surgical candidates, and patients who refuse surgery. Most studies evaluating the efficacy of dCTRT for resectable disease in East have been non randomized studies.^25-33^

The only randomized clinical trial was conducted by Teoh and colleagues.^34^ 81 patients with resectable mid esophageal or distal esophageal SCC were randomized to esophagectomy or dCTRT. There was no significant difference between the two groups with respect to DFS or OS.

Kato and colleagues reported a complete response of 62.2%, a median survival of 29 months, and 5 - year survival of 36.7% after dCTRT for stages II/ III SCC (JCOG 9906).35Although these results are inferior to the standard surgical treatment, it provides a reasonable nonsurgical option for those who wish to avoid or cannot tolerate esophagectomy.

Hence from the eastern perspective, definitive chemoradiation in resectable stages II-III is considered only if patient is not fit for surgery or refuses the surgery.

At the same time in West, based on the results of CROSS trial^22^ (pCR in 29% of patients: 49% for SCC and 23% for AC) has led some to consider a surveillance approach in treating patients with SCC. Two randomized clinical trials addressed this issue in West.

Stahl and colleagues compared dCTRT versus nCTRT plus surgery in locoregional esophageal SCC (cT3-4, N0-1, M0) in the upper/mid third thoracic esophagus.^36^ In their study, 172 patients were randomized to trimodality therapy (chemotherapy followed by nCTRT with 40 Gy followed by surgery) or chemotherapy followed by dCTRT, with at least 65 Gy. The surgery arm had better local control, as evidenced by 2-year progression-free survival of 64% compared with 41% in the CRT arm (P = .003). However, this did not result in improved survival: 2-year OS was 40% for the surgery arm and 35% for dCTRT arm. In addition, treatment-related mortality was significantly higher in the surgery arm (13% vs 3.5%, P = .03). Another important finding from this study is that response to induction chemotherapy was an independent prognostic factor. Subgroup analysis revealed that responders had significantly better prognosis, and that the addition of surgery in this particular group did not change outcome. On the other hand, in nonresponders, those who had complete resection showed improved survival compared with the nonsurgical group.

In the French study FFCD 9102, Bedenne and colleagues randomized 259 patients with T3N0-1M0 esophageal cancer (both AC and SCC) to dCTRT only or nCTRT followed by surgery.^37^ Both groups received CTRT consisting of fluorouracil/cisplatin and 46 Gy radiation. They were then randomized to undergo surgery or continue CTRT. The study population was mainly SCC (89%). No difference in survival was observed: 2-year OS of 34% for the surgery arm versus 40% for dCTRT despite improved local control in the surgery arm. In a subgroup analysis of those who responded to induction chemotherapy, 3-year OS was similar in both groups.

### dCTRT - SALVAGE SURGERY

For patients with residual disease or recurrent disease after dCTRT, salvage surgery is the recommended treatment option in east.^38^ However, surgeons are reluctant often to pursue this approach due to high morbidity and mortality.^39^ Kumagai and colleagues reported a meta-analysis of 4 retrospective studies comparing survival and treatment-related mortality in patients submitted to salvage esophagectomy or second-line chemotherapy for recurrent or persistent SCC after dCTRT.^40^ There was a long-term survival benefit for patients undergoing esophagectomy, with a pooled hazard ratio (HR) for death of 0.42 for salvage surgery compared with second-line chemoradiotherapy (P = .017). However, salvage esophagectomy was associated with a treatment-related mortality of 10.3% in the 36 patients who underwent resection.

In West, The SALV trial assessed the impact of salvage esophagectomy after definitive CRT for esophageal cancers.^41^ Data were collected retrospectively for patients undergoing planned surgery after nCTRT (n = 540) and patients undergoing salvage esophagectomy (n = 308), and further compared patients who benefited from salvage esophagectomy in the setting of persistent disease versus recurrent disease after dCRT.

Both OS and DFS were similar for planned surgery and salvage surgery (3-year OS 43% vs 40%, P = .54; 3-year DFS 39% vs 33%, P = .23). When comparing persistent versus recurrent disease within the salvage group, 3-year OS was better in recurrent disease (56%) compared with persistent disease (41%, P = .046), with a similar trend seen in DFS (3-year DFS 52% for recurrent vs 37% for persistent, P = .095). The study demonstrates that salvage esophagectomy results in acceptable outcomes.

For patients who are deemed to have clinical complete response, the role of surgery also has been questioned. There is an ongoing trial in the Netherlands (the SANO trial) that is randomizing clinical complete responders to either surgery or surveillance.^42^

## NEOADJUVANT CHEMOTHERAPY VERSUS NEOADJUVANT CHEMORADIOTHERAPY

It is clear from the trials that induction treatment is required before surgery in case of cII-III disease though the protocols differ in East and West. There is not a single answer whether neoadjuvant chemotherapy or nCTRT is better. Hence, there are few trials which addressed this issue.

### NeoRes trial^43^

The NeoRes was a randomized trial comparing induction chemotherapy versus nCRT. Patients with resectable SCC and AC, including GEJ tumors (T1-3, any N, M0-M1a, except T1N0, UICC 6th ed) were enrolled. Three cycles of 5-FU + cisplatin chemotherapy was given. Patients who were randomized to receive nCTRT also received 40 Gy. Majority of patients underwent Ivor-Lewis or Mckeown's esophagectomy with few undergoing transhiatal esophagectomy or extended total gastrectomy). In total, 181 patients were randomized; 91 underwent neoadjuvant chemotherapy and 90 underwent nCTRT. nCTRT group had higher pCR rate (28% vs. 9%). Treatment-related complications were similar between the groups although postoperative complications were more severe in nCTRT group. Five-year progression-free survival was 38.9% (95% CI 28.9%-48.8%) in nCTRT group versus 33.0% (95% CI 23.6%-42.7%) in the chemotherapy group, P = 0.82. Five-year OS was 42.2% (95% CI 31.9%-52.1%) versus 39.6% (95% CI 29.5%-49.4%), P = 0. 60. There were no differences in recurrence patterns between the treatment groups. Despite a higher tumor tissue response in nCTRT group, no survival advantages were seen. Consequently, the results do not support unselected addition of radiotherapy to neoadjuvant chemotherapy as a standard of care in patients with resectable esophageal cancer.

### Burmeister et al^44^

Patients with esophageal and GEJ AC were randomized into either chemotherapy with 2 cycles of Cisplatin and Fluorouracil, or nCTRT with the same chemotherapy regimen plus 35 Gy radiotherapy. If patients were without systemic disease at restaging, they underwent esophagectomy. In total, 75 patients were randomized: 36 patients underwent chemotherapy and 39 underwent nCTRT. Rate of R0 resection was higher in nCTRT versus chemotherapy only (100% vs 86%, P = .04). DFS and OS were similar between both groups. The study demonstrated that adding concurrent radiotherapy to preoperative chemotherapy did not increase morbidity or mortality, and did increase R0 resection rates, but not survival.

### Stahl et al^45^

Patients with GEJ AC who had T3 or T4 disease, no prior treatment, and who were surgical candidates were randomized to receive chemotherapy with Cisplatin, Fluorouracil, and Leucovorin in 2.5 cycles, or nCTRT with the same chemotherapy regimen in 2 cycles followed by 3 subsequent weeks of cisplatin and etoposide plus concurrent 30 Gy radiation. In total, 119 patients were randomized: 59 patients received chemotherapy with 52 subsequently undergoing surgery, whereas 60 patients received nCTRT and 49 patients sub-sequently underwent surgery. Rates of R0 resection were similar, although pCR was higher in the nCTRT group (P = .03). nCTRT was associated with a trend toward improved OS (P = .07) at 3 years and less tumor progression on therapy (P = .06) Subanalysis of pathologic nodal status, irrespective of treatment arm, noted an increased 3-year survival for negative nodes (76.5% vs 59.0%, respectively, P<.001). The study demonstrated that adding concurrent radiotherapy to preoperative chemotherapy improved DFS and OS without increase in morbidity, although they note that their sample size limited their ability to provide statistical significance for this finding. Initial results were published in 2009, with updated results published in 2017, as the initial trial did not meet its primary endpoint of survival at 3 years. At this reanalysis, OS still trended toward significance in favor of nCTRT (P = .055), and local progression-free survival remained statistically significant (P = .01).

### Spicer and colleagues^46^

Authors reviewed 3 prospective databases of patients (n=214) with cT3N1 disease undergoing induction chemotherapy versus nCTRT for esophageal and GEJ AC. Patients underwent varying cycles of chemotherapy with a Fluoropyrimidine and Platinum-based agent versus taxane. Patients undergoing nCTRT had the same chemotherapy plus 50.4 Gy of radiation. Surgical resection, via en bloc esophagectomy with D2 and mediastinal lymphadenectomy, plus possible cervical lymphadenectomy based on tumor location, occurred 4 to 6 weeks after therapy. Surgical approaches differed among institutions in terms of fields of lymphadenectomy, thus there were significant differences in the number of patients undergoing 3-field esophagectomy after chemotherapy (39.8%) versus nCTRT (7%). Morbidity and mortality rates were similar between groups, including anastomotic leaks (nCTRT 15% vs chemotherapy alone 11.6%, P = .54). There were no significant differences in OS or DFS, although nCTRT trended toward improved DFS (26.4 months vs 16.0 months, P = .135). This review demonstrated that there is no OS or DFS difference between nCTRT and neoadjuvant chemotherapy, although there is a trend to improved DFS with nCTRT. Number of fields of lymphadenectomy were an independent predictor of worse outcomes. There were interinstitutional treatment differences in surgical approach that must also be considered. Thus, they concluded both treatment modalities are acceptable, so long as en bloc esophagectomy follows neoadjuvant therapy.

### Sjoquist and colleagues, meta-analysis^47^

A total of 24 randomized controlled trials with intention-to-treat were analyzed. Twelve studies compared nCTRT with surgery, 9 compared chemotherapy with surgery, 2 compared induction nCTRT and chemotherapy, and 1 compared all. 4188 patients were included a perioperative chemotherapy regimen per the FLOT protocol with nCTRT per the CROSS protocol. Patients with resectable AC of the esophagus, including Siewert 1 GEJ and some patients with Siewert 2 and 3 with evidence of esophageal infiltration will be included in the study.^50^

## CONCLUSIONS

It is clear from the available trials that an induction across all trials. nCTRT, compared with surgery alone, showed a statistically significant survival benefit of 8.7% over 2 years (P<.0001), both for SCC (P = .004) and AC (P = .02). Induction chemotherapy, compared with surgery alone, showed a 2-year 5.1% survival benefit (P = .005). This benefit remained statistically significant for AC only (P = .01). nCTRT, compared with induction chemotherapy, showed no survival benefit, although both of these trials were underpowered due to early closure. The review concluded that there is a survival advantage for induction therapy (both CRT and chemotherapy) over surgical monotherapy, and that this benefit applies to both SCC and AC. nCTRT did not show any added benefit when compared with induction chemotherapy.

### Ongoing trials

There are few important ongoing trials that may resolve the controversy of superiority of nCTRT or induction chemotherapy. Neo-AEGIS is a randomized trial comparing the 2 established regimens for esophageal and GEJ AC: perioperative chemo per MAGIC regimen Vs induction CRT per CROSS regimen.^48^ TOPGEAR trial is another randomized phase III comparison of perioperative chemotherapy per MAGIC regimen versus the same regimen with the addition of 45 Gy of induction radiation in GEJ AC.^49^ ESOPEC trial is a prospective trial comparing treatment is required for the better outcome in cII-III esophageal cancer. Till the results of ongoing trials become available, CROSS protocol for esophageal SCC and FLOT protocol for AC should be considered the standard of care.
